# Three-Dimensional In Vitro Cell Culture Models for Efficient Drug Discovery: Progress So Far and Future Prospects

**DOI:** 10.3390/ph15080926

**Published:** 2022-07-27

**Authors:** Shaimaa M. Badr-Eldin, Hibah M. Aldawsari, Sabna Kotta, Pran Kishore Deb, Katharigatta N. Venugopala

**Affiliations:** 1 Department of Pharmaceutics, Faculty of Pharmacy, King Abdulaziz University, Jeddah 21589, Saudi Arabia; haldosari@kau.edu.sa (H.M.A.); skotta@kau.edu.sa (S.K.); 2 Center of Excellence for Drug Research and Pharmaceutical Industries, King Abdulaziz University, Jeddah 21589, Saudi Arabia; 3 Department of Pharmaceutical Sciences, Faculty of Pharmacy, Philadelphia University, P.O. Box 1, Amman 19392, Jordan; 4 Department of Pharmaceutical Sciences, College of Clinical Pharmacy, King Faisal University, Al-Ahsa 31982, Saudi Arabia; kvenugopala@kfu.edu.sa; 5 Department of Biotechnology and Food Science, Faculty of Applied Sciences, Durban University of Technology, Durban 4001, South Africa

**Keywords:** 3D cell culture, hydrogel, spheroids, organoid, microfluidic devices, 3D bioprinting, drug repositioning

## Abstract

Despite tremendous advancements in technologies and resources, drug discovery still remains a tedious and expensive process. Though most cells are cultured using 2D monolayer cultures, due to lack of specificity, biochemical incompatibility, and cell-to-cell/matrix communications, they often lag behind in the race of modern drug discovery. There exists compelling evidence that 3D cell culture models are quite promising and advantageous in mimicking in vivo conditions. It is anticipated that these 3D cell culture methods will bridge the translation of data from 2D cell culture to animal models. Although 3D technologies have been adopted widely these days, they still have certain challenges associated with them, such as the maintenance of a micro-tissue environment similar to in vivo models and a lack of reproducibility. However, newer 3D cell culture models are able to bypass these issues to a maximum extent. This review summarizes the basic principles of 3D cell culture approaches and emphasizes different 3D techniques such as hydrogels, spheroids, microfluidic devices, organoids, and 3D bioprinting methods. Besides the progress made so far in 3D cell culture systems, the article emphasizes the various challenges associated with these models and their potential role in drug repositioning, including perspectives from the COVID-19 pandemic.

## 1. Introduction

Drug discovery and development is a lengthy and expensive process due to the high attrition rate in the clinical success of therapeutic agents [1]. To improve drug discovery success rates, newer technologies with higher precision are required. Two traditional and promising approaches in drug discovery include biochemical assays and cell-based assays [2]. Biochemical assays are straightforward and consistent methods to screen out compounds with an expected therapeutic potential towards a target enzyme or receptor [3]. On the other hand, cell-based assays are more complicated and utilised for functional aspects in a cellular framework [4]. Traditionally, cell-based assays were performed in two-dimensional (2D) monolayer cells cultured on various types of planar substrates [5]. The 2D cultures were predominantly used for cell-based high throughput screening to discover drug-like molecules. Currently, these 2D cell models are reliable and very effective approaches for predicting responses of various drugs in vivo as well as for understanding vital molecular and underlying cellular mechanisms [6]. Also, these models have been successfully employed in studying disease pathologies and biomarker discovery [7]. Although, the monolayer models have had vast utilisation in the past, they are still not able to reiterate major in vivo facets, leading to their limited utilization in the modern drug discovery process. Beyond this, the 2D models also have other limitations such as lack of tissue-specificity, mechanical issues, biochemical disturbances, and cell-to-cell/cell-to-matrix-incompatibilities [8,9]. All these issues reveal them to be weaker models to envisage drug efficacy for some specific diseases like cancer. 

**Figure 1:**
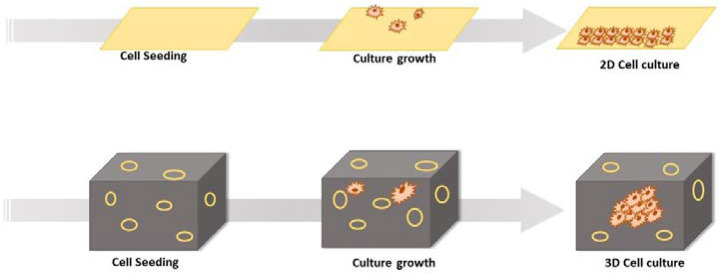
Diagrammatic representation of 2D cell culture and 3D cell culture.

The newer three-dimensional (3D) cell culture techniques have been widely explored in the past decade in drug development, which has led to improved precision and a reduced failure rate of drugs in clinical phases. The accomplishment of 3D-culture models in early drug discovery has been widely adopted nowadays by the pharmaceutical research and development sectors [10,11,12]. It is well established now that 3D-culture systems mimic the tissue factors and are the best representatives of the in vivo cellular phenomena in comparison to 2D models [13,14]. One of the greatest advantages of 3D models is that, together with stem cells or primary cell models, they are able to predict the efficacy as well as toxicity of therapeutic candidates in humans before drugs enter clinical trials [15]. Hence, these are contributing greatly to reducing the attrition rate of drug discovery and development processes. During 3D-culture experiments, as the cell culture mimics the in vivo cellular atmosphere, more efficient observations related to cell-to-cell interactions, tumor properties, metabolomics, stem cell research, and pathophysiology of many other diseases can be studied [16]. In the upcoming sections of this review, we summarize the basic concepts of 3D-culture technology and various associated models with special emphasis on their utilization in drug discovery process. Also, current challenges associated with 3D-culture-based assays and future directions are discussed in brief. Table 1 depicts a comparison of 2D- and 3D-culturing systems (also refer to Figure 1).

## 2. 3D Cell Culture Technologies

**Figure 2:**
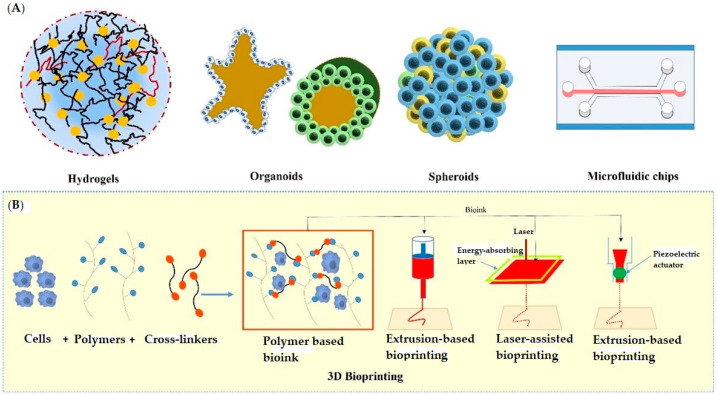
Various approaches used in 3D cell culturing; (**A**) showing hydrogels, organoids, spheroids, and microfluidic chip, (**B**) showing the extrusion-based, laser-assisted, and inkjet-based methods used in 3D Bioprinting.

The past few years have evidenced success stories of development in 3D-culture models that imitate in vivo physiology. These technologies have been widely adopted in cell biology, tissue engineering, as well as in several clinical investigations. The significant 3D approaches include multicellular spheroids, organoids, scaffold approaches, microfluidics/lab on chip techniques, 3D bioprinting, hydrogels, anchorage approaches, hanging drop microplates, magnetic levitation, etc. All these approaches have been utilized by several research groups in drug discovery and development (discussed below). Figure 2 depicts various 3D cell culturing methods.

### 2.1. Hydrogels

Due to their greater veracity, hydrogels have achieved a popular place in ex vivo tissue-like structure cultivation of cells, thereby becoming ideal candidates for 3D culture of tissues due to their similarities with the biological properties of extracellular matrices (ECM) [17]. In general, hydrogels are made up of crosslinked polymers that can absorb large amounts of water (about 95% of their weight) along with other solutes within their swollen matrices, thereby allowing sustained delivery of absorbed solutes. These polymers exhibit desirable properties such as diverse mechanisms, different mesh sizes, puffiness, and deprivations that provide a significant advantage in 3D cell culture models [14]. They can exist as a polymer molecular network due to the presence of intermolecular crosslinks or as fibrillar hydrogels formed via interfibrillar crosslinks [18,19]. The hydrogels can be classified into various categories based on their structural properties, degradability, molecular charge, responsiveness to external stimuli, and source of production [20].

The most common biological source-derived hydrogels used as extracellular matrices proteins are collagens, Matrigel, and alginate. the source of these hydrogels is biological, they are more compatible with 3D cell culturing [21]. The complex nature of these hydrogels makes them non-tuneable, which is disadvantageous in the design of 3D models with required features. To overcome this issue, a number of synthetically produced hydrogels have been reported by various research groups with tuneable material properties as required in 3D cell culturing. 

Sawhney et al. reported poly(ethylene glycol)-based photopolymerized bioerodible macromolecules as hydrogels with successful application in 3D cell culturing [22]. These hydrogels were found to be effective at maintaining encapsulated cell viability, reproducibility, tuneable mechanical properties, and low cost production [23]. Zustiak and co. give PEG derived hydrolytically degradable hydrogels with tuneable, degradable and mechanical properties [24]. Findings from this study suggested that the properties of hydrogels can be controlled by altering polymer density, molecular weight, and the distance between the ester and thiol group in the cross-linker at the same time as keeping hydrogel repeat units and functional groups constant to maintain the cross-linking and degradation conditions compatible for protein and cell encapsulation. Martens et al. reported acrylated poly(vinyl alcohol) macromolecules as hydrogels synthesized via photopolymerization crosslinking [25] and revealed that the choice of crosslinking method greatly influences the polymer network structure in several aspects. Horak and his group successfully employed these hydrogels in a mouse embryonic stem cell model [26]. Notably, polyacrylamide hydrogels matrices posesses easily quantifiable elasticity, which can be modified by adjusting the relative concentrations of the monomer (i.e., acrylamide) and cross-linker (bis-acrylamide). Tse et al. have given a photoinitiated polymerization method for polyacrylamide derived hydrogels with tuneable mechanical properties via varying concentrations of acrylamides due to fabrication [27]. Some other researchers also synthesized hydrogels include poly(ethylene oxide) (PEO), poly(methacrylic acid) (PMMA) [28], poly propylene furmarate-co-ethylene glycol (P(PF-co-EG)) [29,30], poly(acrylamide) (PAAm) [27], poly N-isopropylacrylamide (PNIPAAm) [31], etc. [32]. The low-cost production, consistency, and tuneable properties have made them the centre of attraction in 3D cell culture. In contrast, these synthetic hydrogels are less biocompatible as compared to hydrogels obtained from natural sources due to a lack of endogenous biological moieties [33]. The biocompatibility of synthetic hydrogels can also be enhanced via choosing a compatible starting material, but it can increase the cost depending on the synthetic procedure. 

Considering the potential of electrically conductive nanocomposite scaffolds, researchers aimed to engineer functional cardiac tissue phenotype with enhanced electrical excitability and signal propagation. A group of researchers had designed and fabricated four different gelatin methacrylate (GelMA) hydrogels candidates, including 5% GelMA (mechanically soft), 20% GelMA (mechanically stiff), GelMA-silica nanomaterials (non-conductive with nano-topographies and mechanically soft) and GelMA-gold nanorods (conductive with nano-topographies and mechanically stiff) [34]. It was reported that GelMA-silica nanomaterials and GelMA-gold nanorods hydrogels significantly improved cardiac myocyte adhesion affinity as compared to 5% and 20% GelMA. This highlighted the influence of nano-scale topography exhibited by the nanomaterials on cellular adhesion and retention and promotion of maturation of engineered cardiac tissues. In contrary to the hydrogels comprising of microspheres, Jaklenec et al. [35] fabricated 3D scaffolds from protein-loaded microspheres as the building blocks of scaffold generation for tissue engineering, i.e., poly(lactic-co-glycolic acid) (PLGA) microspheres containing bovine serum albumin. Given the versatility of this simple scaffold fusion method for embedding essentially any combination of loaded microspheres into a 3D structure, it can be used extensively in tissue engineering and therapeutic localized drug delivery. Table 2 represents some of the recently developed hydrogel systems along with their successful applications in 3D cell cultures.

### 2.2. Spheroids

**Figure 3:**
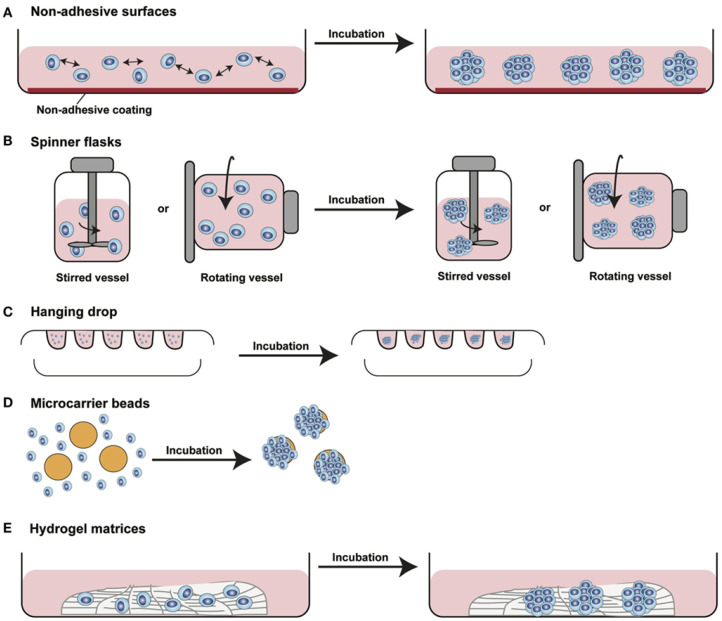
Diagram showing different approaches used to develop 3D spheroids. (**A**) Non-adhesive surfaces: modified culture plates with reduced surface adhesive force allows spontaneous cell aggregation to form cellular spheroids, (**B**) Spinner flasks or gyratory rotator: continuous medium mixing or a constant flask rotation prevents cell adhesion causing massive production of 3D spheroids, (**C**) Hanging drop method: cells suspended in small drops onto the underside of an inverted hanging drop plate induces accumulation of spheroidal aggregates due to gravity forces, (**D**) Microcarrier beads: solid beads of natural or synthetic origins allows surface coating to produce minispheroids that subsequently aggregate to form bigger spheroids, (**E**) Hydrogel matrices: natural or synthetically composed hydrogels are incubated with the cells for their aggregation [This figure is adopted with permission from Manuela et al., 2017 [44]].

Spheroids are cell aggregates that can self-assemble in an environment that usually does not allow adhesion to a smooth surface [36]. Spheroid cultures were first developed around 1970 as multicellular cultures to reiterate the phenotype of human cancer cells and their retort to radiation therapy [37]. After that, spheroid cultures have been utilized for a wide variety of cells, such as stem, hepatic, and neuronal cells. Contrary to the monolayer cultures, 3D spheroids exhibit heterogeneous cell colonies, i.e., cells at proliferating, quiescent, hypoxic, apoptotic, and necrotic stages. The outer layers that are highly exposed to the medium comprise viable and proliferating cells, whereas the core cells tend to be in a hypoxic or quiescent state as they receive less oxygen, nutrients, and other essential compounds from the medium [38]. Also, these have a definite geometry and well-defined cell-to-cell and cell-to-matrix communications. Various membrane (integrins) and extracellular matrix proteins are responsible for the formation of spheroids. Spheroid construction involves aggregation of dispersed cells resulting from long-chain extracellular fibres, allowing binding of surface integrins, leading to upregulation of cadherin expression [39,40]. Further, this cadherin gets deposited on the cell membrane surface, which is responsible for haemophilic cadherin-cadherin interactions, forming tight connections between adjacent cells, resulting in spheroid formation. Finally, the integrins are involved in the activation of focal adhesion kinase (FAK), the overexpression of which has been linked to tumor growth [41,42]. The spheroids have been recently utilized in various drug discovery processes against a variety of diseases, especially in oncology research. Also, the 3D-spheroids can be employed to study the metabolic processes in both intra-cellular and extra-cellular environments; for instance, cardiac 3D-spheroids used to study diseased human heart cells. Importantly, these spheroids further allows the assessment of redox-activity differences between human healthy and dilated myocardium-derived primary mesenchymal cells by scanning electrochemical microscopy, which undoubtedly makes it more convenient to use [43]. Several approaches have been employed to generate the spheroids. The most significant approaches along with their applications have been discussed in the following sections. Figure 3 depicts different methods employed to develop 3D spheroids, particularly to study tumor biology and efficacy of antitumor drug candidates.

#### 2.2.1. Hanging Drop Method

The hanging drop model is a well-known 3D-culture model for spheroid formation. It is a subpart of suspension cell culture techniques developed in the late 1800s [45]. In hanging drop plates, spheroid formation takes place in suspended droplets via self-aggregation under the effect of gravity in bottomless wells [7]. Cells required for spheroid formation are typically suspended in media droplets using micropipettes and segregated below the aperture of the hanging drop plate in bottomless wells within walls (Figure 3C). After segregation in the droplets, these cells eventually form spheroids. The most primary application of the hanging drop culture technique is in embryology. It allows for uniform microtissue formation with reliable results [46]. 

The main disadvantages of the conventional methods associated with hanging drop techniques are that they are labour-intensive and further transfer of micro spheroids is required to other plates for biological assay [16]. The micro spheroids formed under normal conditions might be of various sizes and shapes in hanging drops, which might be overcome by the application of physical external forces [47]. However, use of external forces can significantly affect the biochemical and physiological characteristics of cells in micro spheroids, which can further alter their responses in biological assays [48]. Although recently, several approaches are reported to have overcome these problems with superior quality of results to demonstrate the upper hand of this technique in 3D spheroid formation for creating in vivo-like culture outside the tissue to accelerate drug development. Cho and co. reported the 3D engineerable spheroid formation using a pressure-assisted network for droplet accumulation as the hanging drop method of advancement [49]. The reported method was superior in controlling the size and shape for uniformity with the desired artificial niche as required during spheroid formation. Wu et al. reported a polydimethyl-siloxane (PDMS)-based device with successful application in pumping first cells to droplets followed by a continuous supply of fresh media through those droplets [50]. The device was based on the differences in pressure between reservoirs connected to the microfluidic chip. Huang and his team developed a microfluidic-based hanging drop culture system having a taper-tube design [51]. The reported system provided superior stability of droplets in a cell culture system along with an improved fluid exchange rate. The accumulation of cells at the bottom of droplets in these systems provides convenient methods for observation and analysis. Ware and co. successfully reported the formation of a homogenous 3D pancreatic cancer cell spheroid with the modified hanging drop method [52]. For providing homogeneity to spheroids, methylcellulose polymer was used with highly reproducible results. The method was found successful in all five (Panc-1, BxPC-3, Capan-1, MiaPaCa-2, and AsPC-1) cancer cell lines. Michael et al. reported surface-engineered paper hanging drop chip spheroid formation [53]. The resulting method was successfully employed in spheroid formation along with in situ analysis. This method overcame the disadvantage of transfer of spheroids to other surfaces before analysis, which might be quite helpful in the screening of new molecules at an improved rate. Gao et al. reported modified hanging drop methods with rings to control spreading [54]. The method provided hanging drops with controlled geometry generated by surface gravitational force. The balance between gravity and surface tension can play a key role in defining the geometry of hanging drop spheroids. Similarly, a research group also established that micro-rings can stabilize these droplets for long term culture of spheroids [55].

#### 2.2.2. Magnetic Levitation

In the magnetic levitation technique, magnetic nanoparticles are used to provide a magnetic environment for cells for retain their cellular properties during 3D tissue formation [56]. Souza and colleagues reported the use of gold and iron oxide nanoparticles to cells in the 2D phase, as well as magnetic levitation to preserve cellular features during organoid formation [56,57]. When cells are unattached and suspended in media only, external forces such as magnetic forces can be easily applied for manipulation [58]. The cells further aggregate under a provided environment for organoid formation with extracellular matrix (ECM) with physiological relevance such as collagen [59]. As the ECM and nanoparticles used are physiologically compatible, the cellular functions of cells, such as proliferation, remain unaltered without any inflammatory response. Positive magnetophoresis is the most advantageous over negative magnetophopresis for simulating weightlessness in cells due to its levitating effect only on the surface of cells and does not affect inside cellular functions [60]. Under magnetic levitation, the cells (diamagnetic objects) move to a low magnetic field gradient to result in stable magnetic levitation. The weightlessness simulation is maintained until the gradient is maintained and can facilitate the analysis of fast cellular processes [61,62].

During magnetic levitation, magnetic strength plays a major role in cell culture. The magnetic field of 30–500 G does not affect the cellular functions, but if it is increased to 800–4000 G, it can alter the cellular behaviour to a great extent [63,64]. The strength of the magnetic field can be reduced to a lower value via enhancing the magnetic vulnerability of the medium. In such cases, paramagnetic solutions can be used [65,66,67]. Aside from that, using iron-based nanoparticles in 3D organoids changes the color to brown, which can be advantageous or disadvantageous in diagnostic applications such as IHC with 3,3-diaminobenzidine, MTT assay, and so on [68]. The risk of attaching cells to the plate surface rather than levitating magnetic nanoparticles is also there in this technique. Besides all these limitations, several upgrades are made to improve the magnetic levitation process in 3D cell culture and tissue engineering. For example, the use of low-adhering plates or ultra-low adhering plates can overcome the problem of cell attachment to the surface of plates. 

A research group reported drug screening against breast tumors, cultured using magnetic levitation [69]. The nanoshuttles^TM^ were used for levitation in the presence of a magnetic field. Under a magnetic field, cells with nanoshuttles^TM^ internalised aggregate and form a 3D organoid with controlled density and composition (depending on the density of cells seeded). The technique was successfully applied for in vitro breast cancer tumor formation, mimicking the properties of human in vivo tumors [70]. Tseng et al. used magnetic levitation for the assembly of a three-dimensional multitype bronchiole [59]. The model can be utilized for detecting inflammatory responses in bronchioles and can also help in research related to airway remodelling. In their in vitro 3D breast cancer model [71] Leonard and Godin employed magnetic manipulation. The nanoshuttles^TM^ were successfully used for levitation for co-culture of tumor cells along with fibroblasts or adipocytes and in vitro 3D tissue formation with defined composition and density. 

Tim and co. developed magnetically manipulated 3D structures of HEK293s and SMCs for mobile-based imaging to study toxicity as in wound healing studies [72]. The levitated cells were organized into 3D-structure formation by physical disruption and repatterned into 3D ring structures, which were utilized to determine a ring closure rate. As the toxicity of drugs increases, the rate of ring closure will decrease. Gaitán-Salvatella et al. reported the use of magnetic levitation for 3D osteoblast spheroid formation [73]. The cells aggregated within 24 h of magnetic manipulation to give a microtissue-like structure. The developed microtissue was best suitable for visualization of cell-cell interactions, real-time quantitative PCR analysis, and cell viability studies. Kotze and co. derived a 3D granuloma spheroid based on magnetic levitation [74]. The developed spheroid mimicked the early stage of the tuberculous granuloma spectrum in cellular features. The different manipulations in density of various cytokines or cell numbers can help to understand the infectious pathway behind TB. Another study reported successful covalent immobilization of granulocyte colony-stimulating factor (G-CSF) using magnetic silica gel beads (MagBs) surfaces. Interestingly, the usage of magnetic particles reduced the loss of G-CSF-modified particles, thereby enhancing the outcome of the modification process. However, a higher amount of MagBs-immobilized G-CSF is required to obtain similar efficiency as free G-CSF molecules [75].

#### 2.2.3. Rotary Cell Culture Method

Another promising approach employed for spheroid formation is culturing of cells inside an agitated bottle (Figure 3B). This assembly does not allow the cells to adhere with the substrate and undergo self-assembling. This method is simplest and high yields of spheroids can be obtained through this. However, it can cause mechanical damage to the cells and is also associated with the issues of longevity and variation in size of culture. A modified technique employs a rotating flask along with the horizontal axis, which avoids cell damage under the influence of microgravity and minimal forces, thereby obtaining uniformity in spheroidal size. Recently Cui et al. have utilized rotary cell culture systems (RCCS) to investigate the influence of simulated microgravity on 3D-cultured neural stem cells [76]. The study concluded that as compared to the traditional static cultures, 3D-cultured neural stem cells in RCCS bioreactors exhibited better neuronal differentiation and migratory ability. Most importantly, this study suggested that employing a RCCS bioreactor in combination with a neurotrophin-3 containing medium may be a useful strategy to develop effective neural stem cells for stem cell therapy. In another report, Tang et al. has demonstrated a combination of 3D and rotary culture systems as a promoter of proliferation and differentiation in rat bone marrow mesenchymal stem cells [77]. Rotary cell culture systems can produce rotary movement to imitate microgravity effect by vector-averaged gravity method leading to loss of intracellular metabolic activity response to gravity. This causes altered cell perception of gravity direction and of achieving a dynamic culture system for vital cell movements.

#### 2.2.4. Addition of Nanofibres

Another promising approach for spheroid generation is the addition of polymeric nanofibres, which are being added to a suspension of cultured cells. In 2012, Shin et al. prepared one such system by adding PLGA nanofibers to a cultured suspension of human kidney embryonic cells and dermal fibroblast cells. Nanofibres promote spheroid generation and decrease cell death resulting from a lack of cell adherence [78]. Usually, spheroid formation occurs as a result of collisions between nanofibres and cells within a stirred suspension culture. This interaction between nanofibres and cells is mediated by vitronectin and fibronectin present in the serum medium, which gets adsorbed on the nanofibres to assist cell adherence [78,79]. However, the formation of spheroids in the absence of nanofibres may occur due to the interaction of cadherins of adjacent cells. Recently, Lee et al. have reported stem cell spheroids hybridized with single-segmented nanofibres. The electrospinning method was used to incorporate poly(-lactic acid) single-segmented fibres into these spheroids. Furthermore, the fragmented fibres were coated with polydopamine to increase cell binding affinity, resulting in spheroids of varying sizes. It was found that fibre-containing spheroids were homogenous and increased the cell viability, whereas simple cell spheroids were associated with the loss of DNA, structural degradation, and apoptosis. It was clear that the functions of a spheroid varied with its size. The largest spheroid revealed the greatest angiogenic factor release, whereas the smallest spheroid showed larger effects of osteogenic differentiation [80]. In another report, Rathnam et al. prepared smart hybrid spheroids by adding biodegradable nanofibric materials. The prepared spheroids were capable of deep drug delivery and homogenous 3D cell-matrix interactions. They utilized a spinal cord injury animal model to demonstrate high survival rates, differentiation patterns, and functional recovery of the stem cells. The prepared hybrid spheroids were found to be a substantial system to pave a path for stem cell–based treatment of CNS injuries [81]. Table 3 represents the various techniques of spheroid formulation along with their properties and applications.

### 2.3. Microfluidic Technology

The recent developments in microfluidic technology have made it a tool of great significance in cell culture and assays, with its main application in in vitro 3D-cell culture mimicking the in vivo tissue microenvironment [82]. It is based on the fabrication of small devices (microsized) having microchannels and chambers which control the behaviour of fluids in them. Microfluidic fabrication in 3D-cell culture, which was first developed in the 1980s, offers a number of advantages, including a controlled cellular microenvironment without intrusions from the outside environment, less reagent ingesting, corresponding processing and analysis, and so on [83,84]. The small amount of microfluidics for the fabrication of a small number of cells can result in the production of biomimetic models with in vivo tumor microenvironment [85], different types of cells [86], and biochemical gradients [87]. These simple and less costly models provide efficient and high-throughput screening of drugs at the cellular, organ, and whole-body level. The use of microfluidics provides in situ platforms for drug screening with reproducible results [88]. The use of transparent materials in microfluidic devices can further simplify the direct analysis of various cells based on absorbance or fluorescence [89] and the direct analysis of tagged proteins in microtissues [90]. Gelatin [91] PDMS [92] are most widely used for the design of new microfluidic devices. For instance, a method of fabricating a 3D cell culture system by stacking multiple layers of PDMS embedded with functionalized hydroxypropyl cellulose methacrylate porous scaffolds was reported, which employed thread as a cost-effective transportation channel to overcome diffusion limitation by continuously supplying nutrients and removing waste [93].

The analysis of 3D cultures plays a key role in designing and testing types of microfluidic devices. The type of analysis often decides the selection of microfluidic systems, channel volumes, or dilution factors for efficient results [94]. Bauer et al. reported the use of microfluidic channels with hydrogels for co-culture of breast cancer cells [95]. In continuation of their work, they successfully analysed the F-actins and tubulin proteins in the 3D-cell culture. The results also demonstrated the biomimetic nature of this 3D culture over 2D culture in mobility edges [96]. For example, Mosadegh et al. employed the microfluidic device for fluorescent imaging of lung cancer cells for the determination of cell migration under different oxygen gradients. The 3D culture was done on stacked papers and each layer was analysed after 24 h intervals of exposure to gas and media [97]. Similarly, various drugs or their formulations were also screened for their ADMET properties using microfluidic device-based cell cultures (3D organoids). One such example was the screening of drugs (Cisplatin) on kidney tissue designed using microfluidic devices [98]. The kidney tissue was created on microfluidic channels and then used to study the filtration, reabsorption, and toxic effects of cisplatin on renal cells and nephrons. Jang et al. designed a multi-layered microfluidic device (PDMS microfluidic channels) for developing biomimicking tubular environments. The rat inner medullary cells were cultured in the system which was observed to transport water-soluble protein within cells like in vivo tissue to control water and ion balance [99]. The use of microfluidic devices for the development of tissue-on-chip is well explored today for the screening of drug molecules. Lungs-on-chip was developed using microfluidic technology to study the toxicity of drugs, blockage of airways [100], oxygen transfer efficiency [101], inflammatory effects on lung cells when exposed to various pathogens or nanoparticles [102], and other stress factors [103]. A liver-on-chip model was built to study pharmacokinetic parameters, hepatotoxicity [104], and phase I/II metabolism of drug molecules [105]. Deosarkar et al. developed a neonatal blood-brain barrier on-chip model to study biomimetic nature and permeability as well as the human blood-brain barrier [106]. Jiang et al. created two co-polyester and poly(dimethylsiloxane)-based microfluidic devices for drug molecule screening [107]. Dhiman et al. reviewed recent developments in on-chip tumor models for combinatorial screening of drug molecules [108]. Muscle-on-chip is another application of microfluidic technology to provide a small molecule screening platform against a number of muscular dysfunctions such as myasthenia gravis, muscular dystrophy, mitochondrial myopathy, etc. [109]. All the above-discussed factors describe the key importance of microfluidic technology in 3D culture along with the drug development phase [110]. Table 4 describes the different microfluidic devices with their tissue models and applications in drug screening.

The microfluidic devices are also combined with other methods to further reduce the challenges associated with 3D-cell culturing. The microfluidic devices are used for molding hydrogels into the required shape [111]. Bischel et al. used the hydrogels along with microfluidic techniques. The difference in fluidic properties such as viscosity and pressure helps in creating a vessel inside for the adherence of cells to hydrogel [112]. Wang et al. created a microfluidic device that entraps colon spheroids and suspends them in hydrogel for extracellular matrix support [113]. Derda et al. combined the microfluidic technology with paper cell culture (8 layers of paper) for the culture of MDA-MB-231 cells [114]. The paper provides a diffusion surface for cell secretions, supply of nutrients and growth factors from media to microtissue developed.

### 2.4. Organoids

An organoid is a 3D construct that comprises multiple cell types originating from stem cells by self-organization, which are capable of mimicking the structure and functionality of the native organs [115]. Organoids are basically organ buds, representing an expanding dish-based tissue family showing the exact microanatomy [116,117，^118^]. On the basis of the pattern of their formation, organoids are either tissue organoids or stem cell organoids. Tissue organoids are free from stromal cells and are associated mainly with epithelial cells, which have the inherent capability of self-assembling into tissue-like structures. On the other hand, stem cell organoids originate from embryonic or induced pluripotent stem cells such as neonatal stem cells or resident tissue adult stem cells [119]. There are certain limitations of organoid cultures, such as an unfavourable microenvironment, a lack of interactions with immune cells, and insufficient immune responses. But, organoids originated from human cells possess the potential to establish physiological models to study human development and associated diseases. Organoids can be obtained through several approaches, such as the direct culturing of cells as a monolayer on a stream of feeder cells. An ECM-coated surface can also be used where organoids are formed after differentiation of the cells. Another significant method is using a mechanically supported culture to allow differentiation of primary tissues [120]. An example of such an approach is human keratinocytes, which are capable of self-assembling into fully layered tissue when cultured in air-liquid interface for a few weeks. One more approach is to produce embryoid cells on the plates with low adhesion or via a hanging drop culture. Organoids can also be formulated using serum-free floating cultures of embryoid-like aggregates, which quickly get aggregated on adhesion plates.

Advanced organoid cultures have also provided platforms for drug screening in discovery programs, and in vitro cultures have been well established for various organs. To date, in vitro organoids have been available for the thyroid, pancreas, liver, stomach, intestine, cardiac tissue, cerebral cortex, kidney, lung, and retina. Various types of stem cells and pluripotent cells from animals as well as human beings have been utilized to prepare the organoid systems (refer to Table 5). A wide variety of media/methods such as Matrigel embedding, hanging drop, FTDA, SUVEC, and fluorescent technologies have been used for their preparation. These prepared organoids have opened new paths to study various kinds of pathogenesis of diseases as well as in drug discovery and design. Various reported organoids along with their source, method of preparation, and significant applications have been summarized in Table 5.

Nowadays, paradigms have been shifted towards patient-derived organoids as they offer numerous kinds of advantages [136]. Patient-derived organoids are capable of maintaining chemoresistance and genetic mutations that commonly appear in original tissues [137]. These can be used instead of cancer cell lines, animal models, and in tumor xenografting [138]. These can act as biobanks for drug development, especially in the study of tumors [139]. Recently, organoids have been utilized to predict the treatment responses to radio/immune therapies. There are several advances being made, such as organoids-on-a-chip, which have enhanced the clinical applications of organoids along with the discovery of newer therapeutic candidates.

### 2.5. 3D-Bioprinting Techniques

3D-bioprinting involves printing of cells or biocompatible components into complex tissues by adopting suitable cell frameworks and topologies [140]. By using an additive manufacturing process, biological materials/cells are positioned layer-by-layer into a desired design. Bioprinting can be achieved through three techniques: biomimicry, autonomous self-assembling, and fabrication. Biomimicry involves principles of bio-engineering to achieve replication of cellular/extracellular constituents of any tissue or organ [141]. In the autonomous self-assembling technique, cell-driven histogenesis occurs, which produces the desired micro-framework of functional tissues. In the third approach, small-tissue units are fabricated and assembled into larger blocks, which are prepared by a rational design strategy or self-assembling approach [142,143]. Extrusion-based, laser-assisted, and inkjet-based approaches are examples of modern bioprinting methods (Refer to Figure 2) [144]. A wide variety of bio-inks are available nowadays for the purpose of printing. Various factors like an ink’s flow properties, chemistry, polymeric nature, biocompatibility, viscosity, etc. are considered when selecting a bio-ink. Bio-inks are usually made up of synthetic polymers (e.g., PEGTA, PEGDA) [145,146,147], carbohydrate polymers (e.g., alginate, agarose, gellan gum etc.) [148,149,150] or protein polymers (e.g., collagen [151,152,153,154], fibrin [155,156], Decm [157], GelMA [158,159,160,161] etc.). 3D-bioprinting offers bespoke micro-frameworks, high-throughput screening potentials, as well as subculturing capabilities. This technique is linked to issues such as printing/biomaterials requirements and tissue functional abilities [140]. 3D bioprinting has been used to create a wide range of functional tissues, including skin tissues, bone tissues, respiratory tissues, cardiac tissues, cartilage, and vascular tissues [162]. All these functional tissues have been reported in various transplantation procedures. Beyond this, the generated tissues through this technology can act as excellent models for drug discovery, profiling, and screening [163]. The success stories of applications of 3D-bioprinting have been evidenced by several scientific reports in the fields of therapeutics. For instance, Kundu et al. have developed a new hybrid cartilage substitute comprised of alginate, chondrocytes, and polycaprolactone. Polycaprolactone is advantageous for providing long-lasting stability [147]. Park et al. have prepared a similar kind of autologous cartilage using the same composition as for auricular assembly [164]. Rathan et al. prepared another form of cartilage via functionalizing alginate and cartilage dECM [165]. According to another report, Hung et al. prepared printed cartilage using a biodegradable polyurethane as a bio-ink material. The aqueous solubility of this bio-ink was excellent, which provided ease of mixing with the biomolecules [166]. Duarte et al. have produced a regenerated cornea in a dome shape similar to the original cornea by using agarose and collagen bio-ink and printed keratocytes. The regenerated cells showed good viability and the same features as the original keratocytes and the same kind of transparency as that of real cornea [167]. In another report, Kim et al. have developed cornea-specific lamellae by printing keratocytes encapsulated in the cornea using dECM bio-ink. Cell alignment was kept vertical, resulting into similar lattice structure as that of the original cornea [168].

3D-bioprinting technology has also been successfully employed for the regeneration of skeletal and cardiac muscles. Kang et al. have printed muscle structures using a mixture of gelatin, fibrinogen and hyaluronic acid as bio-ink and encapsulated the C2C12 myoblasts [169]. Choi et al. utilized dECM bio-ink to print vascularised muscular structures using the coaxial nozzle method. This coaxial method provided benefits such as improved voluntary muscle loss recovery, vascularisation, and contraction recovery [170]. Gaetani et al. carried out printing of human fetal myocardial cells using a mixture of hyauronic acid and gelatin as bio-ink. They fabricated the heart patches to assist the availability of oxygen and nutrition [171,172]. In another significant work, Jang et al. printed human c-kit+ cardiac cells with vascular endothelial growth factor utilizing heart dECM bio-ink. The 3D printing was achieved through an extrusion-based methodology for treating the mouse myocardial infarction model. The outcomes of this work were improved vascularisation and improved myocardial functions [173].

Beyond the therapeutic applications, 3D-bioprinting techniques have been successfully employed in the generation of drug screening models as these are excellent alternatives to animal models. Various drug screening models such as liver, kidney, skin, and cancer have been reported for the screening of drugs. For instance, 3D-printed metastatic in vitro models for cancer were established by Meng et al. with spatially positioned cells, growth factor release reservoirs and other biomaterials. They utilized laser irradiation technology to copy the tumor metastatic properties and angiogenesis. Further, the utility of this 3D-cancer model was revealed by testing the efficacy of immunotoxins to provide a platform for drug screening [174]. Another model was developed by Cui et al. for breast cancer metastasis to bone using optimized bio-ink for tumor, endothelial, and osteoblast cells. SLA 3D printing technology was utilized, enabling the study of endothelial migration along with colony formation of cancer cells [175]. Bhise et al. have developed an in vitro hepatic model using 3D bioprinting by using a photocurable gelatin-based bio-ink. This model was then further used to study the hepatic toxicity of acetaminophen [159]. Another liver-on-a-chip model was established by Lee et al. using compartmental fragments of hepatocytes in a single step process using gelatin bio-ink. This hepatic model revealed an improvement in the viability of the cells and the synthesis of urea and albumin [154]. Neil et al. have prepared a 3D kidney model of a vascularized proximal tubule. They made use of fugitive bio-ink made from pluronic F127 and poly-ethylene oxide. Further seeding of renal tubular epithelial cells and glomerular endothelial cells was done in the microchannels. The prepared model was capable of nutrient exchange and reabsorption of materials between proximal tubules and blood vessels [176]. 

Due to the ban on animal testing for cosmetic products, 3D skin bio-printing has come into fashion to replace the conventional models of skin product testing. A significant skin model was put forward by Lea et al. They utilized a bio-ink comprising of gelatine, alginate, and fibrinogen to produce dermis derivatives and printed human dermal fibroblasts by seeding epidermal keratinocytes onto the dermis layer. It was evident that the morphology of the created skin model was similar to that of the original human skin after 26 days [177]. 

Various applications of 3D-bioprinting in therapeutic areas as well as in drug screening models have been summarized in Table 6.

## 3. Role of 3D Cell Culture Models in Drug Repositioning

3D cell cultures have been well established models for drug discovery, disease modelling, drug testing, and toxicity analysis in comparison to conventional 2D models [178]. Beyond this, the pattern studies of transcriptional factors’ expression and receptor behaviour are implemented by 3D-culture models, which have a great application in drug repositioning and repurposing [179]. Drug repositioning is a technique that uses the therapeutic value of an existing drug by targeting ailments other than the one for which it was originally approved [180,181]. A combination of microarrays, bioinformatics, and 3D-culturing models is an excellent approach for drug repositioning [182]. 3D-cell culture induced gene expression has paved a new path for drug discovery and drug repositioning. A prominent study reported 3D-cell-culture-induced gene expression changes in human neuroblastoma cells by analyzing 1766 genes using microarray analysis performed through RT-PCR reaction [183]. Some gene expression changes were noted, and it was concluded that several changes in features of cultured cells resulted from varied gene expression. In another study, microarray analysis performed over 9600 genes by smooth muscle cells. It is worth noting that 77 genes were expressed more than twice in 3D-cultured cells. The enhanced cyclin-dependent kinase inhibitor 1 and decreased tyrosine phosphorylation of adhesion kinase observed in 3D-cultured cells were significant indicators of drug repositioning [184]. Tsunoda et al. reported a potential application of non-malignant prostatic cells in the study of prostate cancer biomarkers beyond the study of genes associated with prostate cancer [185]. Yin et al. reported the study of multidrug-resistance-hepatotoxic effects of methotrexate in rats along with the multidrug resistance caused by the MRP2 gene [186]. Pruksakron et al. have studied targets associated with nucleotide metabolism along with mitochondrial and proteins associated with aerobic glycolysis [187]. Breslin et al. studied differential responses of targets to drugs, modified expression of targets associated with drug resistance in human breast cancer-cell lines along with the study of proteins and enzymes associated with anticancer drugs [188]. Horning et al. have studied the surface-engineered 3D cultures of breast cancer-cell lines and studied the effects of anticancer drugs on them. Along with this, they studied the significant discrepancy in the action of drugs and the associated factors with it [189]. In another study, Loessner et al. studied the effects of paclitaxel on bioengineered hydrogel cultures of ovarian cancer epithelial-cell lines along with the study of drug-resistance patterns in the cells [190]. Nirmalandhan et al. have prepared collagen gel-cell cultures of human lung cancer-cell lines to study the activity of anticancer drugs. They also studied various alterations in the action induced by the drugs [191]. 

Recently, a group of researchers fabricated 3D-lung-cancer organoids by using a pleural effusion aspirate and most importantly by incorporating cells obtained from the patients directly to enable personalized disease modelling and tumor characterization [192]. Interestingly, the isolated patient cells-derived organoids demonstrated anatomically relevant structures and exhibited cancer-specific characteristics that enabled comparative assessment of chemotherapy responses. Another group has reported a 3D cell-based phenotypic assay that determined the effects of radiation and ten established chemotherapeutics in radiation-resistant breast cancer cells grown in 3D-microtissue spheroids [193]. In this study, heterotypic cultures of normal human dermal fibroblasts and three mammary cancer-cell lines (T47D, MDA-MB-231, and MDA-MB-361) were used to recapitulate the complexity of mammary cancer. Of the ten drugs analysed, vinblastine was found to be more effective, when concurrently given with radiation therapy. A novel in vitro 3D-printed fluidic device that allows nutrient exchange and the diffusion of toxic metabolites from the spheroids to outside was also reported [194]. Notably, MALDI imaging MS revealed that prodrug irinotecan (a chemotherapeutic agent) penetrated into the tumor spheroids and localized into the center of the spheroid (in the necrotic core), and its metabolite SN-38 was concentrated on the outside region (representing the capability of the cells to metabolize the prodrug). Undoubtedly, this finding supports that the model is efficient enough to mimic the in vivo conditions and can be used to assess drug penetration and metabolism in cancerous cells. A similar finding was reported in 3D-multicellular tumor spheroids used to analyse the distribution of irinotecan as measured by serial trypsinization and nanoflow liquid chromatography-tandem mass spectrometry [195].

The coronavirus disease 2019 (COVID-19) pandemic, caused by the severe acute respiratory syndrome coronavirus 2 (SARS-CoV-2), recently wreaked havoc around the world, prompting a push for faster drug discovery and development [196,197]. Among the different 3D-cell-culture techniques, organoid models (followed by microfluidics-based platform) have been widely considered for COVID-19 research, including alveolar lung organoids, hPSC-derived airway organoids, adult bronchial organoids, hPSC-derived kidney organoids, hPSC-derived liver organoids, brain organoids, etc.. A detailed explanation of these models can be found at [198,199]. Nevertheless, as compared to the animal models, organoids still exhibit certain limitations due to the lack of blood vessels or vasculature, immune cells, and interorgan communication. 

Table 7 represents certain 3D-culture model types and their potential role in drug repositioning.

## 4. Conclusions

3D-cell-culture techniques have emerged as a great tool of interest in drug discovery and toxicity predictions across a wide range of biological indications. The utilization of 3D-cell-culture techniques is rapidly expanding due to the development of new models providing the same results as in the case of complex in vivo methods. The ability of 3D models to recapitulate in vivo systems has provided a significant advantage in drug development and reduced the burden on animals. Currently wide study associated with 3D models lies within academia, only focusing on the development of more biorelevant models. There are huge hurdles which need to be crossed to take them to industrial levels. For this purpose, more compatible, biologically relevant, cost-effective and reproducible 3D-culture models need to be developed. Although daunting challenges such as development of biologically compatible 3D cultures and cost-effective and biomimicking environments in microtissues lie ahead in the development of new models, the list of 3D-cell-culture models is increasing daily. The combination of biomedical engineering with disease mechanisms can provide more relevant methods with more information on particular phenotypes associated with disease conditions and help in particular with precise findings. The review of the above methods and their applications clearly dictates that 3D-cell-culture models hold distinct promise in drug development, high-throughput screening, disease or cell-based analysis, cell interactions, etc. With the expanding culture of use of 3D models, it is just a matter of time before these 3D models provide a major breakthrough in drug development in more complex diseases/disorders.

## 5. Expert Opinion and Author’s Perspective

The transition from 2D-cell culture to 3D-cell culture technique can provide significant advances in drug development and reduce the likelihood of failure in later clinical stages, but it is also associated with significant concerns. The first issue is the application of 3D-cell-culture models in high-throughput screening [200]. In drug development, high-throughput screening is the first step in the screening of a large number of libraries for desired biological activity [201]. The cost of 3D in vitro models in high-throughput screening and reproducibility of results is a major concern. Scalability of 3D models to multi-well microplates and compatibility with currently established assay methods are also major challenges in the application of these models in the screening of large numbers of compounds [202]. The hanging drop model is the most common method employed in microplate readers, but it still needs a lot of expertise for applicability in high-throughput screening. Slight variations in the culture of 3D models can produce some modifications in permutations which can affect the reproducibility of results in them. Poor light penetration and light scattering in 3D cultures present a major obstacle in imaging techniques [203,204]. Evidence suggests the successful use of different microscopic techniques, including scanning electrochemical microscopy, atomic force microscopy, and confocal fluorescence microscopy in the assessment of cell growth at both 2D and 3D conditions [43,205,206]. However, fluorescence microscopy presents a major challenge in the application of 3D models as the recording of the *z* coordinate with fixed imaging at xy coordinates becomes quite difficult along with extended time consumption and low magnificent results. The 3D models such as the hanging drop model are quite incompatible with fluorescence microscopes. Application of 3D organoids in flow cytometry is another major concern as it becomes an end-point technique. In flow cytometry, before cell sorting and biomarker detection, the organoids/spheroids need to be converted to single-cell suspensions, after which they become useless and need to be disposed of [207]. The selection of matrices for brain-specific organoids or barriers is another challenge in this transition [208]. The level of physiological oxygen provided in such models is still a source of conflict in the scientific community. Colom et al. studied various acellular or cellularized extracellular matrices in which sparse gels were not found to be suitable surrogates for 3D organoids [209]. The denser gels also provide a lower physiological oxygen level to cells in these models. 

Although shifting from 2D-culture to 3D-culture techniques is still in a transition phase with a number of obstacles in the way, it can provide more efficient and successful results in the drug development process. A number of research groups have commented on the shift from 2D-culture to 3D-organoid models for screening of drug molecules [7,204]. Recently, de Bournonville and co. reported the successful testing of a benchtop bioreactor developed for controlled environmental conditions, self-regulated 3D progenitor cell cultures, and bioprocessing effectively [210]. 3D organoids can provide more effective and efficient methods for organ transplantation. It can also provide better patient-derived tumor models for drug development purposes [211]. 

The replacement of animal-derived constituents such as serum, laminin, collagen, and membranes used in cell cultures hinders the consistency and produces complications. The filters used in 3D organoids such as alginate, foam, and most microcarriers are best suited to provide an animal-free environment and overcome these obstacles [209]. With more research groups shifting toward 3D models, it will help to overcome the shortcomings in current models. It will help to develop more advanced, easy to handle, compatible, cost-effective and efficient models for clinical studies. The more in vivo-like conditions provided via these organoids will reduce the burden on animals and reduce the cost of the drug development process.

## Figures and Tables

**Table 1:** A comparison of 2D cell culture and 3D cell culture.

2D Cell Culture	3D Cell Culture
Limited physiological relevance	Better than 2D cell culture in physiological relevance
Culture formation occurs within a few minutes to a few hours	Culture formation takes a few hours to a few days
High performance, simplicity of culture, and easy to interpret	Compromised performance, complexity of culture, and difficult to interpret
Does not mimic the tissue environment	Mimics the in vivo conditions of tissues and organs
No cell-cell and cell-extracellular environment interactions	Proper cell-cell and cell-extracellular environment interactions
Altered morphological characteristics and cell division process, thus loss of polarity and phenotype	Preserves morphological characteristics and cell division process, thus diverse polarity and phenotype
Changes in mRNA splicing, gene expression, topology and cellular biochemistry	mRNA splicing, gene expression, topology, and cellular biochemistry are representative of in vivo environment
Homogenous distribution and unlimited access to essential compounds (contrasting the in vivo conditions)	Heterogenous distribution and variable access to essential compounds (similar to that of in vivo conditions)
Poor drug metabolism	Good drug metabolism
Inexpensive	Comparatively expensive due to the requirements of some expensive materials and special equipment
Reproducibility is feasible	Reproducibility is difficult

**Table 2:** Recently reported advanced synthetic hydrogels.

Sl. No.	Starting Material	Synthetic Method	Properties	Application	Ref.
1	Poly(ethylene glycol)	Crosslinking of PEG vinyl sulfone (PEG-VS) with PEG-diester-dithiol	Hydrolytically degradable hydrogels with tuneable, degradable and mechanical properties	Balb/3T3 fibroblast adhesion and 3D matrices	[24]
2	Poly(2-hydroxyethyl methacrylate)	Radical polymerization	Open porous structures with voids of the size and shape of crystallites	Mouse embryonic stem cell model	[26]
3	Polyacrylamide	Photoinitiated polymerization	Tuneable mechanical properties	--	[27]
4	Poly(methacrylic acid)	Green fabrication (Emulsion polymerization)	pH responsive hydrogel	--	[28]
5	Poly propylene furmarate-co-ethylene glycol	Covalently linked RGD cell-adhesive peptide	Macroporous, mineralized	Differentiation of marrow stromal cells (MSCs)	[29,30]
6	Poly N-isopropylacrylamide	Polymerization	PNIPAAm gel partially occupied with chitosan pores	3D stem cell culture, Tissue engineering	[31]

**Table 3:** Recent applications of various methods employed in spheroid formulation.

Sl. No.	Method of Spheroid Formation	Technique/Model Utilized	Properties	Application	Ref.
1.	Hanging Drop	Pressure-assisted network for droplet accumulation	Uniformity in size and shapes, desired artificial niche, fast and economical	3D glomerulus-like heterogeneous microtissues	[49]
2.	Hanging Drop	Polydimethyl-siloxane (PDMS) based device working on basis of pressure differences	Injection of cells to droplets followed by continuous supply of fresh media inside droplets	Mouse embryonic stem cell culturing for embryonic body formation	[50]
3.	Hanging Drop	Microfluidic-based hanging drop culture system with the design of taper-tube	Increased stability of droplets, enhanced rate of exchange of fluid	Mesenchymal Stem Cell Culture	[51]
4.	Hanging Drop	Methylcellulose polymer based modified method	Homogenous spheroid, Reproducible	Homogenous 3D pancreatic cancer cell spheroid	[52]
5.	Hanging Drop	Surface-engineered paper hanging drop chip	In-site analysis, time-dependent detection of secreted protein, and fluorescence staining without disturbing the spheroids	Paper might be next high-throughput 3D spheroid-based “body-on-a-chip” platform material	[53]
6.	Hanging Drop	Fabricated hanging drop method	Controlled geometry with uniform diameter	β-TC-6 cell spheroids with optimized diameters	[54]
7.	Magnetic levitation	Nanoshuttles™, the Bio-Assembler system and Breast tumor model for drug screening	Large-sized model, Controlled tumor cell composition and density	Drug screening in cancer	[69]
8.	Magnetic levitation	Nanoshuttles™ for co-culture of cells and multitype bronchiole 3D model	Organized 3D cocultures with maintained phenotype	Inflammatory response angiogenesis, airway remodeling research	[59]
9.	Magnetic levitation	Nanoshuttles™ for magnetic manipulation with combination of cancer cells, fibroblasts, myofibroblasts, immune cells or adipocytes	Defined cellular composition and density	Drug screening in cancer, Toxicity measurement	[71]
10.	Magnetic levitation	Nanoshuttles™ assembly for 3D culture and HEK293s, SMCs 3D structures for wound healing studies as in 2D studies	Magnetically manipulated 3D ring type structures for determination of ring closure rate	Toxicity measurement	[72]
11.	Magnetic levitation	Iron oxide (Fe_2_O_3_) and gold (Au) nanoparticles with 3D Osteoblast Spheroid	Real time PCR analysis, visualization of cell-cell interaction in spheroid formation	Tissue engineering	[73]
12.	Magnetic levitation	Nanoshuttles™ for levitation of alveolar macrophage 3D Granuloma Spheroid	Large-sized model, controlled tumor cell composition and density	Study of disease forming cellular functions	[74]
13.	Rotary cell culture	mRNA/miRNA sequencing using luciferase assay and western blot	Expression of NTRK3 was elevated in neural stem culture on collagen sponge culture system	Study of neuronal differentiation and migratory ability of neural stem cells	[76]
14.	Rotary cell culture	Amplification of rat bone marrow mesenchymal stem cells (BMSCs) followed by high-throughput microarray analysis	Rotary cell culture was able to enhance cell proliferation and colony formation, as well as maintain the differentiation	Promotion of proliferation and maintenance of differentiation of rat BMSCs	[77]
15.	Nanofibre addition	Electrospinning to form poly(ι-lactic acid) single-segmented fibers containing spheroids of different sizes	Spheroids of varying sizes by modulating the amount of cells and fibers (0.063–0.322 mm^2^)	To study effect on cell viability and stem cell differentiation	[80]
16.	Nanofibre addition	Biodegradable nanopolymer addition followed by spinal cord injury animal model	Spheroids presented high survival rates, controlled differentiation, and functional recovery	To study the stem cell-based treatment of CNS injuries	[81]

**Table 4:** Applications of microfluidic devices in drug screening.

Sl. No.	Tissue Model/Cell Type	Microfluidic Device	Application	Ref.
1	Kidney-on-chip	Multi-layered PDMS-based microfluidic device	Cell viability, drug screening, transport of protein	[99]
2	Lungs-on-chip	Silicon wafers by spin coating SU-8 2100 negative photoresist-based device	Permeability studies, oxygen transfer efficiency	[101]
3	Liver-on-chip	Elastomeric PDMS stencil devices	Hepatotoxicity, phase I/II metabolism study	[105]
4	Blood-brain barrier on-chip	PDMS-based devices	BBB permeability and electrical resistance measurement	[106]
5	--	Copolyester and poly(dimethylsiloxane)-based different devices	Screening of small molecule libraries, food contaminant analysis	[107]
6	On-chip tumor models	Various microfluidic devices	On-chip combinatorial drug screening	[108]

**Table 5:** Contribution of some reported organoids.

Sl. No.	Organoid	Source	Method of Preparation	Application	Reference
1.	Thyroid	Mouse embryonic stem cells	Hanging drop method	Treatment of hypothyroidism	[121]
2.	Intestinal	Human pluripotent stem cells	Differentiation into definitive endoderm using Matrigel	Studies of human intestinal development and disease	[122]
3.	Lung	Adult mice stem cells	Co-culturing of endothelial cells utilizing Matrigel	Identification of targets in lung diseases and mechanism of respiratory diseases	[123]
4.	Lung	Mice and human alveolar epithelial and fibroblast cells	Fluorescence activated cell sorting, clonal alveolar organoid assays	Identification of new targets for human lung regeneration	[124]
5.	Pancreas	Mouse embryonic pancreatic progenitors	Matrigel 3D culturing	Expansion of pancreatic progenitors to discover cellular therapy of diabetes	[125]
6.	Pancreas	Human pluripotent stem cells	Growth factor-reduced Matrigel and FTDA medium embedding	Modelling of pancreatic diseases and screening for disease-rescuing agents	[126]
7.	Liver	Mice liver GR5^+^ stem cells	Matrigel 3D culturing	Generation of functional hepatocytes, model generation for antitrypsin deficiency and Alagille syndrome	[127]
8.	Liver	Human induced pluripotent stem cells	Co-culturing with HUVEC media and Matrigel embedding	Generation of functional human liver from pluripotent stem cells	[128]
9.	Kidney	Human embryonic stem cells and pluripotent stem cells	Subculturing at air-liquid interface	Kidney organoids generation with nephrons associated with a collecting duct network surrounded by endothelial cells	[129]
10.	Kidney	Human pluripotent stem cells	Culturing by sandwiching between two Matrigel layers	Human epithelial disease modelling and regenerative medicine applications	[130]
11.	Prostate	Human prostrate luminal and epithelial lineages	Serum free conditioned medium with Matrigel embedding	Study of prostate diseases, biology and drug discovery against prostate cancer	[131]
12.	Stomach	Adult stem cells or gastric glands	Matrigel 3D culturing	Studies of gastric epithelial renewal, inflammation, infection and cancer	[132]
13.	Retina	Human embryonic stem cells	Serum-free floating culture of embryoid-like aggregates and Matrigel embedding	Formation of optic cup structure and retinal structures	[133]
14.	Brain	Human pluripotent stem cells	Matrigel 3D culturing with sequence addition of growth factors	Study of self-organizing potentials of polarized cerebral tissues	[134]
15.	Thymus	Fibroblasts	Induced reprogramming by transcription factor forkhead box N1	Generation of entire organs by utilizing cellular reprogramming and use of thymus implantation to boost up immune system	[135]

**Table 6:** Applications of 3D-bioprinting in tissue regeneration, drug screening and drug repositioning.

Sl. No.	Tissue/Model	Bio-Ink Used	Method of Preparation	Application	Reference
1.	Cartilage	Alginate, polycaprolactone	Additive manufacturing	Cartilage tissue engineering and regenerative medicine	[147]
2.	Autologous cartilage	Polycaprolactone	Multihead tissue building system	Auricular reconstruction	[164]
3.	Cartilage	dECM	Layer-by-layer fabrication by multihead discovery system	Regeneration of musculoskeletal tissues	[165]
4.	Cartilage	Biodegradable polyurethane	Low-temperature fused deposition manufacturing	Cartilage tissue engineering and customized tissue transplantation	[166]
5.	Cornea	Agarose and collagen mixture	Drop-on-demand bioprinting	Clinical study of stromal corneal diseases	[167]
6.	Cornea	dECM bio-ink	Shear stress induced fabrication	Corneal tissue engineering	[168]
7.	Human scale tissues	Mixture of gelatine, hyaluronic acid and fibrinogen	Integrated tissue organ printer based on fabrication	Production of human scale tissues with improved integrity	[169]
8.	Skeletal muscle	dECM bio-ink	Co-axial nozzle spray	Generation of biomemetic engineered muscle to treat voluntary muscle loss	[170]
9.	Myocardial cells	Hyaluronic acid and gelatine mixture	Bioscaffolder tissue printing	Preservation of cardiac functions after myocardial infarction	[171,172]
10.	Human c-kit+ cardiac progenitor cells	dECM bio-ink	Extrusion-based technology	Enhancement in cardiac functions and cardiac repair	[173]
11.	Metastatic cancer model	-	Laser irradiation	Creation of vascularised tumor models for drug screening of immunotoxins	[174]
12.	Breast cancer model	Gelatin and PEGDA	Stereolithography	Investigations of breast cancer metastasis to bone	[175]
13.	Hepatic spheroidal model	Photocurable methacryloyl bio-ink	Liver-on-chip platform using fabrication in bioreactor	Assessment of hepatic toxicity of the drugs	[159]
14.	Liver-on-a-chip model	Gelatin bio-ink	One-step fabrication	Development of organ-on-chip systems	[154]
15.	Vascularized renal proximal tubule model	Pluronic F127 and poly-ethylene oxide	3D fabrication	In vitro studies of renal function, disease modelling, and pharmacology	[176]
16.	Human skin model	Mixture of gelatin, alginate and fibrinogen	3D-skin object printing	Development of human skin	[177]

**Table 7:** Potential role of 3D cell culture models in drug repositioning.

Sl. No.	3D Cell Culture System	Primary Application	Application for Drug Repositioning	Reference
1.	Micro-dissected tissues of non-malignant prostatic cells	Prostate cancer associated with RWPE-1 and TA1 genes	Study of prostate cancer biomarkers	[185]
2.	Gel entrapped culture of hepatocytes	Study of MRP2 gene expression	Study of multidrug resistance and evaluation of new drug combinations	[186]
3.	Collagen-based scaffold culture of HepG2 cell lines	Proteins of mitochondria and aerobic glycolysis	Targets in nucleotide metabolism	[187]
4.	PolyHEMA scaffold culture of HER2-positive breast cancer cell lines	Study of anti-cancer drugs, associated proteins and enzymes	Study of differential responses to drugs, increased expression of targets involved in drug resistance, metabolism	[188]
5.	Surface-engineered breast cancer cell lines MCF7	Study of action of tamoxifen, doxorubicin, paclitaxel etc.	Decreased anti-proliferative activity of the drugs	[189]
6.	Gel-entrapped culture of human hepatoma cells	Study of methotrexate	Study of increased drug resistance and modulation through hormones	[186]
7.	Hydrogel matrix of human ovarian cancer cell lines	Paclitaxel	Resistance for anticancer action	[190]
8.	Collagen gel-based cultures of lung cancer cell lines	Paclitaxel, doxorubicin, cisplatin, gemcitabine	Alterations in drug-induced activity	[191]

## Data Availability

Data sharing not applicable.
